# Social Cognition Dysfunctions in Neurodegenerative Diseases: Neuroanatomical Correlates and Clinical Implications

**DOI:** 10.1155/2018/1849794

**Published:** 2018-04-26

**Authors:** Foteini Christidi, Raffaella Migliaccio, Hernando Santamaría-García, Gabriella Santangelo, Francesca Trojsi

**Affiliations:** ^1^First Department of Neurology, Aeginition Hospital, Medical School, National and Kapodistrian University of Athens, Athens, Greece; ^2^INSERM U 1127, CNRS UMR 7225, Sorbonne Universités, and Université Pierre et Marie Curie-Paris 6, UMRS 1127, Institut du Cerveau et de la Moelle Épinière (ICM), 75013 Paris, France; ^3^Pontificia Universidad Javeriana Bogotá, Colombia, Centro de Memoria y Cognición Intellectus Hospital Universitario San Ignacio, Bogotá, Colombia; ^4^Department of Psychology, University of Campania “Luigi Vanvitelli”, Caserta, Italy; ^5^Department of Medical, Surgical, Neurological, Metabolic, and Aging Sciences, University of Campania “Luigi Vanvitelli”, Naples, Italy

## Abstract

Social cognitive function, involved in the perception, processing, and interpretation of social information, has been shown to be crucial for successful communication and interpersonal relationships, thereby significantly impacting mental health, well-being, and quality of life. In this regard, assessment of social cognition, mainly focusing on four key domains, such as theory of mind (ToM), emotional empathy, and social perception and behavior, has been increasingly evaluated in clinical settings, given the potential implications of impairments of these skills for therapeutic decision-making. With regard to neurodegenerative diseases (NDs), most disorders, characterized by variable disease phenotypes and progression, although similar for the unfavorable prognosis, are associated to impairments of social cognitive function, with consequent negative effects on patients' management. Specifically, in some NDs these deficits may represent core diagnostic criteria, such as for behavioral variant frontotemporal dementia (bvFTD), or may emerge during the disease course as critical aspects, such as for Parkinson's and Alzheimer's diseases. On this background, we aimed to revise the most updated evidence on the neurobiological hypotheses derived from network-based approaches, clinical manifestations, and assessment tools of social cognitive dysfunctions in NDs, also prospecting potential benefits on patients' well-being, quality of life, and outcome derived from potential therapeutic perspectives of these deficits.

## 1. Introduction

Social cognition refers to a wide range of cognitive capacities elicited by, about, and directed towards other people [1]. In particular, these skills allow humans to both understand themselves and interact with and understand others, engaging in appropriate goal-directed behaviors [1]. Given that social cognition may play a prominent role in clinical care of most psychiatric and neurological illnesses [2], including neurodegenerative conditions, an emerging literature addresses the study of the neurobiological processes underlying social interactions and the behavioral correlates of the breakdown of these processes. In particular, growing evidence suggests that neurodegenerative diseases (NDs) are associated with some level of social cognitive impairment that has the potential to disrupt interpersonal relationships, thereby eliminating the benefits that social interactions may have for patients with other neurocognitive impairments. However, the frequency, extent, and clinical correlates of these abnormalities are not fully established.

This review aims to summarize some of the basic components of social cognition, also referring recent hypotheses derived from network-based approaches, and to discuss clinical manifestations of social cognitive dysfunctions in most NDs, addressing the pertinent literature published in the last 10 years.

### 1.1. Social Cognition, Social Behavior, and Social Functioning

The term “social” implies that the processing demands are related to specific classes of stimuli, such as emotional expressions on a face, in the voice, or from body posture, also including higher-order functions, such as making inferences about other people's mental states (e.g., theory of mind (ToM)), making moral decisions, regulating emotions and feelings, and experiencing and expressing empathy [1, 3, 4]. Moreover, at the outset, it is useful to clarify the significance of social behavior, cognition, and functioning, which are substantially related to one another.

“Social cognition” refers to any cognitive processing (e.g., perception, reasoning, memory, attention, motivation, and decision-making) that appears to be relatively specialized for the social domain. It causes “social behavior” that comprises the readily observable interactions between an individual and other people, while “social functioning” is broader than social behavior in that it consists in the long-term and contextualized ability of an individual to interact with others (i.e., social behavior when integrated over time and context) [5]. Finally, the “social brain” historically refers to a number of brain structures, some of which, when damaged, may be involved in the impairment of social cognition and behavior (i.e., ventromedial and dorsomedial prefrontal cortex, temporoparietal junction and superior temporal cortex, insula, and fusiform gyrus) [3], while others have been found activated in healthy brains when people perform social tasks in a magnetic resonance scanner by using functional magnetic resonance imaging (fMRI) [1, 3, 6]. Furthermore, considering that no social process can be attributed to a single structure alone, the recent network-based approach to brain function, principally related to the growing implementation of resting state fMRI (rs-fMRI) studies, allowed the identification of a distributed network supporting social function, which included regions from the original social brain [1, 6, 7].

### 1.2. Network-Based Approach to Social Cognition

A core social cognition network is centered on the amygdala which has been proven to play a pivotal role in emotion processing, from triggering emotional responses to detecting socially salient stimuli and performing social affiliative behaviors [4, 8, 9], and to be connected to multiple brain regions involved in emotion circuits [10, 11]. Abnormalities within this network, comprising most components of the social brain (i.e., medial prefrontal cortex, orbitofrontal cortex (OFC), anterior cingulate cortex, temporoparietal junction, inferior frontal gyrus, and superior temporal sulcus), have been revealed in patients affected by schizophrenia [12, 13], frontotemporal lobar degeneration (FTLD) [14, 15], and autism spectrum disorder [16-18]. In particular, activation in the OFC and ventromedial prefrontal cortex has been shown to be not essential for affective responses, but critical for the attribution of meaning to an affective stimulus [19], while activation in the lateral part of the prefrontal region has been found to be associated with a feeling of displeasure and inhibits behavior [20]. Therefore, alterations in these areas may lead to inappropriate social behaviors [21, 22].

A second network involved in social cognition is the so-called “mentalizing” network, which includes the right temporoparietal junction as a key region [23], found to be activated when a subject spontaneously tracks others' mental states [24, 25] and when mentalizing is required as part of another judgment, such as in the case of moral judgments [26] or even when a subject observes a scenario in which a protagonist holds a false belief [27, 28, 29]. In this regard, while ToM refers to the cognitive ability to infer and reason about our own and other people's beliefs, intentions, or emotions, empathy consists in a basic perceptual capacity of understanding others' feelings and subjective psychological states [30], motivating prosocial behaviors [31]. The empathy network, the third circuit implicated in social cognition, includes cingulo-insular structures [32] and has been shown to be impaired in several neuropsychiatric conditions [33, 34]. Finally, of interest for social interactions is also the so-called “mirror neuron system”, mainly involving the inferior frontal gyrus, the inferior parietal lobule, the fusiform face area, and the superior temporal sulcus [35, 36], activated during the observation of the actions of others, including emotion recognition [37, 38], and, therefore, typically impaired in autism [39-41].

### 1.3. Clinical Assessment of Social Cognition

From a clinical point of view, failures of social cognition, most often characterized by impairment of one or more of the four networks cited above, have been assessed using more specific tools useful to investigate the four different domains. Evidence underlined the need for introducing validated tasks of social cognition in the assessment of patients with neurodegenerative diseases [42-44]. In particular, some neurological disorders, characterized by the neurodegeneration of frontomedian areas, such as the behavioral variant frontotemporal dementia (bvFTD), need more specific neuropsychological testing of social cognition for assessing frontomedian cortex functions, in contrast to the well-known sensitivity of executive tests mainly for the frontolateral cortex [45].

With regard to social cognitive measures designed to detect abnormal social behaviors, a range of informant-rated measures (i.e., patients' self-report data might be distorted because of the lack of emotional insight), such as those derived from the Frontal Systems Behavior Scale (FrSBe) [46] and Frontal Behavioral Inventory (FBI) [47], both exploring changes in personality and behavior that are associated with frontoexecutive dysfunction, and from Socioemotional Dysfunction Scale (SDS) [48], Social Inappropriateness Scale [49], and Social Impairment Rating Scale (SIRS) [50], focused on the detection of interpersonal phenomena and social impairment.

With regard to assessing ToM abilities, several tools allow exploring a patient's ability to infer what others are thinking and feeling, and to reason about how their thoughts and feelings will influence their behavior. False-belief tasks [51] are extensively validated measures of ToM that assess the ability to disregard one's own knowledge about the world and consider that someone else might have a different, erroneous belief.

Measures that assess social inference, such as the ability to detect sarcasm, also provide insight into the potential difficulties related to social interaction, such as The Awareness of Social Inference Test (TASIT) [52] that allows the detection of sincerity. ToM alterations can also be assessed with the Strange Stories test [53], in which patients are asked to demonstrate their understanding of stories in which characters' behavior can be best understood by attributing to him/her a specific underlying mental state. The Faux-Pas Test [54] also involves a series of written stories, but patients are asked to detect the *faux pas* and to understand beliefs, intentions, and inappropriateness. Finally, the Reading the Mind in the Eyes Test (RMET) [55] explores the ability to make inferences on the basis of observable features, such as facial expression and eye gaze, asking participants to infer the mental state of a person on the basis of a photograph of their eyes and the surrounding area.

To provide potential insights into empathic disturbances, valuable information may be derived from self-rated and informant-rated measures of affective empathy, such as the Empathic Concern subscale of the Interpersonal Reactivity Inventory (IRI-EC) [56], which investigates the feelings of warmth, compassion, and concern for others; the Perspective-Taking subscale of the IRI (IRI-PT), which allows distinguishing between affective abnormalities that reflect a lack of caring and those that reflect a lack of understanding; and the Empathy Quotient (EQ) [57], which measures the ability of understanding and predicting other people's affective and cognitive empathy and the nature of any emotional response to other people. Finally, among emotion-relevant performance tasks, which consist in evaluating emotional response to viewing photographs or videos, the Multifaceted Empathy Test (MET) [58] allows differentiating between mental state understanding (cognitive empathy) and subjective emotional response (affective empathy).

Deficits of social perception may be manifested as a deficit in identifying others' emotions, and this dysfunction may be assessed through the presentation of static photographs of high-intensity facial expressions. In this regard, the most commonly used task is the Ekman Faces, the one for assessing emotion labelling and discrimination [59]: participants are asked to identify which emotion is shown by a picture of a face and whether two faces show the same or different emotions. Moreover, to evaluate the intensity of a facial expression, the “Facial Expressions of Emotion: Stimuli and Tests” includes images that vary in their emotional intensity, enabling clinicians to create tasks that are graded in difficulty. The breadth and specificity of difficulties in recognizing emotions can be assessed with batteries of tests, such as the Comprehensive Affect Testing System [60] and the Florida Affect Battery [61], which use not only visual stimuli, but also auditory. Both of these batteries incorporate multiple subtasks that assess the ability to process visual (facial expressions), auditory (prosody), and visual-auditory (simultaneous facial expressions and prosody) emotional information.

The evaluation of the ability to integrate social perceptual cues with contextual information that forms part of normal social encounters can also be clinically useful. One measure that can be used for such an assessment is the Emotion Evaluation Test, which forms part of TASIT Part 1 [52], evaluating the ability to recognize emotions from dynamic, multimodal stimuli that are embedded into specific social scenarios. In particular, participants are shown videos in which an actor interprets one of seven basic emotions, sometimes with ambiguous dialogue, sometimes without any dialogue and they are asked to identify the emotional expression depicted.

In summary, when social cognitive dysfunction is suspected on the basis of clinical evidence, at least one measure of each of the four social cognitive domains should be evaluated [5] and, if specific social cognitive deficits are identified, a more comprehensive assessment that focuses on the domain(s) in question should be conducted. In this regard, Table 1 summarizes the abovementioned assessment tools, reporting the neurological disorders in which impairment of social cognition domains has been explored and the respective neuroanatomical correlates of these dysfunctions. The selection of the more appropriate protocol should be guided by the clinical validity of the tests to be administered (i.e., sensitivity and specificity for the neurological disorder of interest) and by the existence of population norms [5], and potential disadvantages may be linked to the lack of population norms in the case of some measures.

## 2. Social Cognition Abnormalities in Neurodegenerative Disorders

### 2.1. Amyotrophic Lateral Sclerosis-Frontotemporal Disease Spectrum Disorders (ALS-FTSD)

#### 2.1.1. Frontotemporal Lobar Degeneration (FTLD)

Social cognition has been reported as selectively vulnerable in FTLD, a term that grouped a clinically and pathologically complex spectrum of non-Alzheimer neurodegenerative disorders featured by selective and progressive atrophy of frontal, insular, and temporal brain lobes [62]. In the late 19th century, this complex group of disorders was denominated as Pick's disease in homage to Arnold Pick who helped in the study and description of these disorders. Although FTLD is considerably uncommon compared to Alzheimer's disease, this disease spectrum is one of the most important causes of young onset dementia and entails high clinical and socioeconomic costs [63].

Three major FTLD clinical syndromes, described considering the predominant clinical manifestations, are the bvFTD, mainly featured by disturbances in social behavior and in executive functioning [64, 65]; the primary progressive aphasias (PPAs) (semantic variant (svPPA), nonfluent agrammatic variant (nfvPPA), and logopenic variant), a group of disorders mainly characterized by linguistic and behavioral alterations [66]); and the syndromes characterized by the cooccurrence of FTLD with neurological disorders mainly affecting cortical and subcortical brain areas, including amyotrophic lateral sclerosis (ALS) [67], progressive supranuclear palsy (PSP), and the corticobasal syndrome (CBS) [63], which mainly affect motor functions, but also impact social behavior.

*(1) Alterations in the Four Domains of Social Cognition.* Social cognition deficits are pervasive in FTLD. However, among the aforementioned syndromes, dysfunctions in social interaction processes have been mostly described in patients with bvFTD [64, 65]. In particular, six core symptoms are recognized in the revised diagnostic criteria [64]: (i) early (i.e., within the first three years of symptom onset) behavioral disinhibition, for example, socially inappropriate behavior, loss of manners or decorum, or impulsive actions; (ii) early apathy or inertia; (iii) early loss of sympathy or empathy, for example, diminished response to other people's needs and feelings and diminished social interest; (iv) early perseverative, stereotyped, or compulsive/ritualistic behavior, for example, repetitive movements and stereotypy of speech; (v) hyperorality and dietary changes, for example, altered food preferences, binge eating, and oral exploration of inedible objects; and (vi) executive dysfunction; with at least 3 of these 6 features required for a diagnosis of bvFTD. In bvFTD patients, impairments have been described in different social cognitive processes associated with previous behaviors ranging from basic affective to more high-order and reflexive processes [65, 68-71] and, interestingly, bvFTD has been proposed as a disease model for studying interactions between emotion processing, social cognition, and interoception [72]. In particular, with regard to basic social cognitive processes, bvFTD patients can exhibit emotion-processing alterations, including abnormalities of the perception of emotional and social cues [73-76], altered empathic concern [77], and alterations in affective expression that includes the presence of apathy or, by contrast, euphoric mood states, overfamiliarity, jocularity, and silliness [78, 79]. Furthermore, the bvFTD patients may present disturbances in the more reflexive social cognitive processes including reduced theory of mind abilities [43], mentalizing deficits [80], diminished prosocial sentiments [81], and reduced long-term cooperative behaviors [70, 82, 83]. Recently, impaired performances at RMET have been revealed to better discriminate bvFTD patients from healthy subjects or Alzheimer's disease patients than altered performances in executive tests, thereby underlining the relevance of social cognition abnormalities in bvFTD diagnosis [84-89]. In particular, executive function tests, such as Stroop task and Trail Making Test, have been shown to be less disease specific than social cognition tests, such as the RMET, for the differential diagnosis across different forms of dementia [85-89]. In support of this, recent meta-analyses confirmed the central role of ToM in bvFTD diagnosis by showing significantly higher and domain-specific impairments of ToM (and emotion recognition) in bvFTD in comparison to control subjects and Alzheimer's disease [90] or other clinical conditions including multiple psychiatric, neurological, and developmental disorders [91]. However, questionnaires that also account for behavioral disorders, such as apathy evaluation scale (AES) [92] and FrSBe scale, especially in the informant-report version, have been shown to significantly differentiate bvFTD patients from healthy controls [84]. Of note, some of the most frequent behavioral symptoms in bvFTD, including apathy, impulsivity, and disinhibition, have been associated to implicit difficulties in social interaction and alterations in the processing of social cues, suggesting a tight interplay between social cognition and neuropsychiatric symptoms in this condition [68, 93]. Thus, the apathetic presentation of bvFTD includes patients who have a lack of interest in their social surroundings as they present difficulty in initiating, planning, and motivating social behavior, related to atrophy in frontal areas and basal ganglia [93-95]. Along this vein, impulsive and disinhibited bvFTD patients exhibit inappropriate social behavior including undue familiarity, disorganized behaviors, and sexual acting out related to impaired mechanisms of cognitive control as a consequence to atrophy in the OFC, frontal ventromedial, and cingulate cortices and anterior temporal areas [96].

Alterations in the social cognitive process can also transfer to moral domains in bvFTD patients, who may show altered moral judgments, displaying more utilitarian judgments in the face of moral dilemmas [97, 98], a pattern also observed in extreme criminal terrorists [99]. Moreover, these patients can display increased antisocial and criminal behavior [100, 101], as well as a relatively high incidence of legal violations [102] and a heightened expression of counter-empathy emotions such as envy and gloating for others' misfortunes [103].

With regard to the ability to make judgements about others' behavior, attitudes, and emotions (i.e., social perception and empathy domains), patients with bvFTD may experience impaired emotion recognition, empathy, and sarcasm detection [73, 77, 104, 105]. In particular, performance on the newly developed TASIT-S, regarding emotion recognition and sarcasm detection, has been revealed impaired in bvFTD and relatively intact in AD [14]. However, although most studies have focused on the verbal categorization of facial expressions [106], deficits have also been reported under different task conditions and stimulus modalities including vocal [107], bodily [108], and musical [109] expressions of emotion. Moreover, the emotion recognition of film stimuli is also impaired [75, 110], although the psychological reactivity to negative film stimuli does not appear to differ from controls [75]. In addition to these deficits in recognizing emotions in others, bvFTD patients have also shown abnormal emotion suppression, emotion generation, and experience of self-conscious emotion, as revealed for bvFTD patients viewing disgust-invoking stimuli, who have been shown to display reduced facial expressions of disgust, reduced physiological reactivity, and reduced self-reported experience of disgust, compared to controls [111]. Furthermore, a deficit in social context processing was observed in the performance of FTLD patients in the empathy for pain task (EPT) [77], a suitable instrument that evaluates empathy in the context of intentional/accidental harm. Accidental pain situations are less clear and explicit; hence, they require greater demands to ascertain the action's intentionality and integration of contextual information. When performing the EPT, the FTLD patients do not easily discriminate between accidental and intentional situations revealing difficulties in integrating social context cues and agents' intentionality [73].

With regard to linguistic variants of FTLD, namely PPAs including svPPA and nfvPPA, some evidence also revealed alterations in several social cognitive processes. Several reports showed deficits in face and emotion recognition, and in ToM processes in both svPPA and nfvPPA [109, 112-114].

*(2) Neuroanatomical Bases of Social Cognition Impairment in FTLD and Network-Based Approaches.* Most of the aforementioned behavioral alterations might reflect a general disturbance in different neural networks. To date, it has been reported that the functioning of three neural networks can be altered in FTLD, including the salience network, the dorsal attention network, and the default mode network. Firstly, the salience network (SLN) [115] composed of the anterior cingulate, insula, striatum, and amygdala, which is activated in healthy subjects during tasks requiring attentional selection, task switching, and self-regulation of behavior, has been reported as impaired in FTLD as a consequence of atrophy over the main hubs of SLN [115]. In particular, the insula, a key region of SLN, is highly connected with the anterior portion connecting with the lateral OFC, while the posterior portion connects with the superior temporal cortex; in bvFTD, both the ventral (frontoinsular) and dorsal areas of the anterior insular are affected [116]. Degeneration of these connected areas has been shown to be related to the impairment of emotion recognition and processing [109, 117], social cognition [118], and interoception [119] in bvFTD.

Secondly, it has been reported that in FTLD there is an abnormal increased connectivity in other networks including the dorsal attention network and default-mode network (DMN) [120]. Alterations in connectivity patterns of those networks seem to be at the core of the decline in executive functions and attention, as well as apathy in patients with FTLD [120, 121]. Thirdly, clinical alterations in FTLD have been associated to a disorder of functional frontolimbic disconnection leading to a compensatory hyperconnectivity in prefrontal areas in response to the absence of affective feedback during the planning and execution of behavior [121].

More recently, an integrative model suggested that the functioning of a network known as the social context network (SCN), composed by fronto-temporo-insular areas, might explain the social cognitive, executive, and behavioral alterations in FTLD [68, 122-124]. Arguably, in control subjects, the SCN favors (a) to update context cues to make predictions, (b) to consolidate context-target associative learning, and (c) to coordinate internal and external milieus [68, 122].

In linguistic variants, comparative analysis of regional gray matter related to social cognition deficits have revealed a differential pattern of fronto-insulo-temporal atrophy in bvFTD, in contrast to a set of dissociable insulo-temporal areas for svPPA and for nfvPPA [112]. Thus, face and emotion recognition impairments in nfvPPA were related to the atrophy of the bilateral posterior fusiform gyrus, bilateral insular cortex, and anterior temporal lobe [112]. Conversely, emotion recognition deficits in svPPA have been associated to atrophy in left temporal structures and also to amygdala atrophy [125]. Finally, deficits in ToM in nfvPPA have been associated with temporal pole and insular cortex degeneration. In contrast, theory of mind disturbances in svPPA are consistent with the patients' atrophy in the left temporal lobe and the medial frontal cortex [113].

Taken together, the integration of the study of social cognitive factors, such as emotion processing, empathy, ToM, moral cognition, and sociobehavioral regulation, are considered as the current target to assess complex behaviors in FTLD. In fact, a deep comprehension of the neurocognitive processes that subsume the interplay between the sociomoral cognition, the executive function, and the behavior seems to be the most robust way to create new perspectives for the diagnosis and new targets of intervention in FTLD.

#### 2.1.2. Amyotrophic Lateral Sclerosis and Its Disease Spectrum

The presence of impaired social cognition in ALS, with or without dementia, provides additional evidence in favour of the existence of an ALS-FTLD continuum and appears to have crucial implications for patients' and caregivers' training from early disease stages. ALS, the most common motor neuron disease, has been traditionally classified as a disease of the motor system. However, cognitive and behavioral dysfunctions are now recognized as an integral part of ALS-related clinical syndrome [67, 126]. In particular, about 50–60% among ALS patients may develop frontotemporal dysfunctions [67, 127], mostly characterized by executive and language impairment, variable memory dysfunctions, and/or behavioral impairment [67, 128]. Apathy is the most pronounced ALS-related behavioral change [129], while disinhibition and disorganization have been less frequently reported [130]. In addition, approximately up to 15% of ALS patients will either present with or develop FTLD, exhibiting a strong clinical and pathological overlap between ALS and FTLD [131]. Thus, the abovementioned heterogeneity results in different categorization groups across a spectrum of disease, including cognitive impairment, behavioral impairment, and ALS-FTLD [67, 131].

Impaired social cognition is now recognized as a part of the cognitive phenotype of ALS, despite the fact that there is significant heterogeneity in tasks used to study social cognition. During the last decades, an increasing body of studies focus on patients' performance in tests related to social cognition [67] and also evaluated its neuroanatomical correlates [132]. On the other hand, social cognition is included in an ALS cognitive screening testing (i.e., Edinburg Cognitive and Behavioral ALS Screen (ECAS); [133]) and in the “Axis II: Defining the neuropsychological deficits” of the recently suggested diagnostic criteria for ALS-frontotemporal spectrum disorders (ALS-FTSD) [67]. Moreover, the evaluation of social cognition in ALS-FTSD may have an utmost importance in clinical settings, given the potential effects of its impairment on patients' quality of life and ability to engage in end-of-life decisions [134-136]. However, it is still debatable whether social cognition deficits are independent of other cognitive deficits in ALS or are part of the executive deficits or not [137-143] and to what degree they are associated with other cognitive deficits, including memory function [136]. Of note, a subgroup of ALS patients without dementia has been found to present impaired social cognition without executive dysfunction [140].

*(1) Alterations in the Four Domains of Social Cognition.* Changes in emotion-processing ability and reduced capacity in the emotional recognition of facial expressions (i.e., mostly related to negative emotions, including fear, anger, and disgust [144, 145]) is more likely in patients with ALS-FTLD [141, 146-148]. In particular, the latter, in association with the fact that the severity of social cognition deficits is much more pronounced and widespread in FTLD patients [149, 150], corroborates the existence of a considerable clinical overlapping between ALS and FTLD.

Social behavior dysfunction in ALS mainly includes apathy [141]. Moreover, loss of empathy [141], deficits in emotion processing [147, 151-153] and emotional empathy attribution [139], and compromised ability to make social inferences [142, 146] have also been described in nondemented ALS patients.

Patients with ALS show difficulty on tests tapping onto ToM components, exhibiting impaired abilities (i) to describe the intentions and feeling of characters [139, 142, 154], (ii) to identify and explain social faux pas [155], and (iii) to estimate preferences for objects based on the interpretation of eye gaze direction [141, 156].

Even though previous evidence revealed a more pronounced impairment in affective rather than cognitive component of ToM [157], Trojsi et al. [136] found that both cognitive and affective ToM may be impaired in the early disease stages by simultaneously comparing both ToM components [136]. Of note, cognitive ToM impairments have been mostly linked to a more general executive dysfunction [154].

Clinical variables have been directly or indirectly related to patients' ToM and other social abilities. For instance, the majority of studies with impairments include patients with an average duration of 30 months [141, 142, 155], bulbar onset [156], and/or cognitive impairment [142, 146]. With regard to the latter, it is still unclear whether social cognitive impairment is independent of other cognitive deficits (particularly executive dysfunction) or not. In particular, Watermeyer et al. [143] revealed that impaired social cognition in ALS has been mainly attributed to primary executive dysfunction, found to be the main predictor of social cognition performance above and beyond demographics, behavior, mood, and personality variables. Severe deficits in both cognitive and affective ToM have been proven to be related to apathy and impaired verbal fluency and naming [157], while early in the disease course impaired ToM has been associated to executive dysfunction [137]. On the other hand, there are several studies that failed to find an association between social cognition/ToM and executive impairment [137, 141, 155]. In this regard, Trojsi et al. [136] recently reported that both cognitive and affective ToM components are associated with nonexecutive impairment, including memory prose and visuospatial ability.

*(2) Neuroanatomical Bases of Social Cognition Impairment in ALS.* Some multimodal studies have directly addressed in vivo the degeneration of social brain networks in ALS, using neuroimaging techniques tailored to the study of structural changes (gray matter, white matter) [139, 148] and functional alternations (fMRI) [132]. Evidence was related to the emotion circuits, including amygdala and medial prefrontal, OFC and anterior cingulate cortices, and “mentalizing” and “empathy” networks. In particular, patterns of gray matter atrophy in anterior cingulate and right frontoinsular cortices were found significantly associated with emotional and empathy performances in nondemented ALS patients [139]. Moreover, a significant decline of emotion recognition skills (particularly affecting the identification of negative emotions) has been found related to microstructural changes (measured through fractional anisotropy) in right inferior longitudinal fasciculus and inferior frontooccipital fasciculus in nondemented ALS patients [148]. Interestingly, also in ALS patients the right hemisphere has been confirmed to play a key role in the identification of others' emotions, especially those negative, with specific damage of ventral associative tracts connecting frontal, temporal, limbic, and occipital areas [147].

Focusing on the cognitive ToM component, Carluer et al. [137] detected significant correlations between cognitive ToM deficit (i.e., false-belief task) and brain metabolic rate of glucose consumption in the bilateral dorsomedial and dorsolateral prefrontal cortex, as well as in supplementary motor areas. These findings are in line with the involvement of dorsomedial and dorsolateral prefrontal areas in cognitive ToM [158, 159], as well as the contribution of the supplementary motor area in the “mirror neuron system” [160].

### 2.2. Parkinson's Disease and Parkinsonisms

Parkinson's disease (PD), mainly characterized by motor symptoms (i.e., resting tremor, bradykinesia, rigidity, and postural instability), may exhibit early nonmotor symptoms, including neuropsychiatric symptoms, sleep problems, and cognitive deficits, hypothesized to be secondary to the loss of dopaminergic neurons in the substantia nigra and the consequent hypostimulation of the prefrontal cortex [161]. Among nonmotor domains, social cognition has been explored in PD, especially with regard to emotion recognition and ToM abilities. However, emerging evidence has underlined the severity of nonmotor symptoms of PSP and CBS, which may substantially impact on social interactions and contribute to alter emotion recognition [162, 163].

#### 2.2.1. Alterations in the Four Domains of Social Cognition

As for emotion recognition in PD, several studies [164-169] revealed emotion recognition deficits in PD patients when compared to matched healthy controls. However, other studies failed to find these deficits [170-174]. A recent meta-analytic review [175], which investigated the emotion recognition from faces and voices in PD, revealed significant and modest deficits of this ability in nondemented PD patients, independently from the level of motor disability. Furthermore, several studies revealed that PD patients were more impaired in recognizing negative emotions (anger, disgust, fear, and sadness) than positive ones (happiness, surprise) [176], while other studies suggested that the recognition of negative emotions may be impaired mainly in the early stages of PD and, then, this impairment has been shown to mainly affect the positive ones [177]. In particular, Hipp et al. [178] showed that, at the early stages, PD patients might be still prone to compensate the deficient input of low contrast sensitivity that is crucial for the appreciation of negative facial emotions.

Impairments of facial emotion recognition in PD patients were found to be independent of depressive symptoms [167, 179-181], executive deficits [179, 180], and clinical aspects (i.e., disease duration and severity, [180]). Moreover, some studies revealed that emotion recognition abnormalities may occur after subthalamic nucleus stimulation [182-185], probably due to alterations of projections to cortical areas, particularly the OFC, which has been already implicated in emotion recognition [186]. However, a recent study of Albuquerque et al. [187] did not confirm these findings in advanced PD.

With regard to PSP and CBS, also belonging to the FTLD spectrum of neurodegeneration (i.e., abnormal function/levels of the microtubule associated protein tau), patients affected by PSP may exhibit impaired facial (i.e., sadness and sadness) and voice emotion recognition [188, 189] as well as CBS patients, who may exhibit difficulties in recognizing disgust, sadness, surprise, and happiness, but not anger and fear [163]. Moreover, half of the PSP patients reported that social impairments negatively impacted their quality of life [190] and, in this regard, this self-perceived social impairment may be the result of the loss of emotion knowledge or breakdown of higher-order social inferences, known as “theory of mind,” as observed in FTLD [105]. In support of this overlap of social cognition impairment between FTLD and PSP, Shany-Ur et al. [191] assessed socioemotional comprehension, including visual perspective taking, belief representation, and emotion reading in a population of neurodegenerative patients, including patients with FTLD and PSP, using the Social Inference-Enriched (SI-E) and Social Inference-Minimal (SI-M) subtests of the TASIT [52]. They revealed that both patients with bvFTD and with PSP had significantly poorer scores than healthy controls on the TASIT SI-E “think” questions across verbal cue items, indicating an impaired ability to represent others' verbalized opinions/beliefs, and on the TASIT SI-E “do” questions across all items, indicating impaired ability to comprehend others' intentions. In particular, impairment of the comprehension of insincere communication and sarcasm was observed in PSP as well as in bvFTD patients, though to a major extent in patients with bvFTD [191]. Of note, failure to comprehend complex social interactions has been demonstrated to exacerbate patients' poor social self-monitoring and aberrant social behavior, thereby severely impacting interpersonal communication and patients' management [192]. Conversely, in CBS observation of facial apraxia, which results in the inability to express facial emotional expressions [193], and flat aprosodic speech [194] may reflect a compromised ability to express emotions.

Several studies explored the two different subcomponents of ToM (i.e., affective and cognitive subcomponents) in PD patients and revealed deficits of both across the entire disease course [169, 195-198]. Importantly, Peron et al. [199] found no different performance on ToM tasks between medicated and nonmedicated PD patients at early stages, suggesting that ToM deficits could be observed in PD patients when the degenerative process has spread beyond the dopaminergic pathways, but not in the early PD patients, in whom neuronal loss is limited to the nigrostriatal and mesolimbic dopaminergic systems. However, more recent studies did not confirm these findings, but revealed the occurrence of impairment of cognitive ToM in both medicated and unmedicated PD [196, 200], whereas other studies showed impairment of affective ToM [195] or deficits of both ToM subcomponents [198, 201].

Deficits of the cognitive ToM subcomponent have been found associated predominantly with executive dysfunction [198, 200, 202-204]. Conversely, Roca et al. [196] did not find any association between cognitive ToM, depression, executive dysfunctions, and medication usage. With regard to deficits of the affective ToM subcomponent, they have been associated with apathy [198] and reduced quality of life [201] and may be predicted by poor cognitive status and the dysfunction of visuospatial abilities [205].

#### 2.2.2. Neuroanatomical Bases of Social Cognition Impairment in PD and Parkinsonisms

With regard to structural neural bases of the deficits in facial emotion recognition, Ibarretxe-Bilbao et al. [206] revealed an association between these deficits and reduced volume in OFC, associated to the degeneration of OFC and amygdala. More recently, Baggio et al. [207] confirmed the pivotal role of abnormalities within these areas in impaired facial emotion recognition and also found that poor sadness, disgust, and anger identification were also related to dysfunction in other cortical regions, such as postcentral and right occipital fusiform gyri, ventral striatum, subgenual cortex, and anterior cingulate cortex.

**Figure 1:**
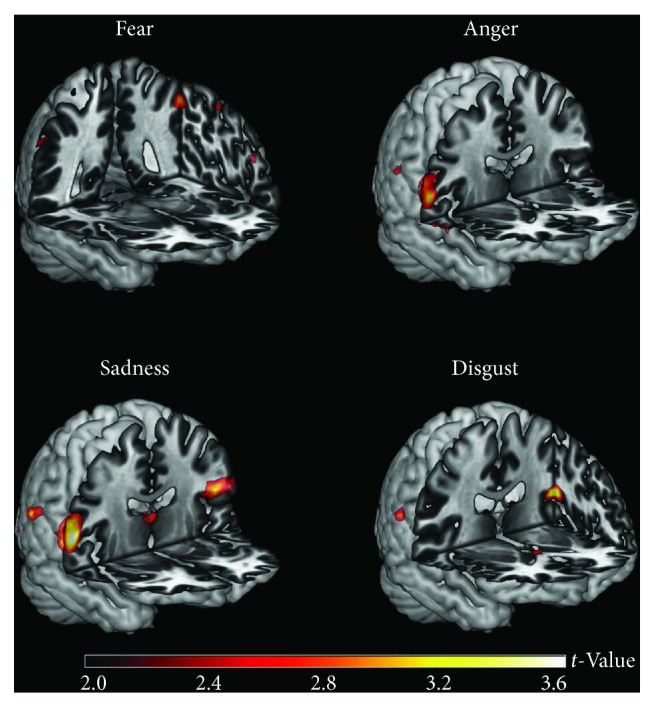
Regions showing increased fMRI activation in nonmedicated, nondemented PD patients (*n* = 17) compared to healthy controls (*n* = 22). PD patients may exhibit a general stronger recruitment of parietal regions when observing pictures of facial expressions depicting disgust, fear, sadness, and anger (image reproduced from Wabnegger et al. [209] under the Creative Commons license (CC-BY), no permission needed).

Previous neuroimaging studies, focusing on functional changes associated with the impaired recognition of emotions in PD patients, revealed that the impaired emotional facial recognition network was characterized by a decreased metabolism within the bilateral posterior cingulate gyrus (BA 31), right superior frontal gyrus (BAs 10, 9, and 6), and left superior frontal gyrus (BAs 10 and 11) [208]. Furthermore, Wabnegger et al. [209] found that, when compared to healthy subjects, PD patients showed a stronger activation in somatosensory cortices, which are involved in decoding emotional states by internally generating somatosensory representations that simulate how one feels when displaying a certain facial expression and, therefore, may be substantially involved in emotion recognition (Figure 1).

With regard to parkinsonisms, impaired emotion recognition in PSP patients have been associated with gray matter atrophy in the right inferior frontal gyrus [188], while in CBS neuroimaging analyses revealed that emotion-processing deficits were associated with the atrophy of the paracentral gyrus/precuneus region, as well as of the basal ganglia [163]. PSP patients have been proven to exhibit mild but significant focal deficits in social cognition [191], which is consistent with evidence showing that they may often manifest behavioral and personality changes, hypothesized to occur as a result of a disconnection between subcortical structures and the prefrontal cortex [210, 211].

With regard to neural correlates of ToM deficits in PD, Péron et al. [212] revealed a significant association between impaired ToM abilities and decreased cerebral glucose metabolism in brain areas belonging to the “ToM network” (i.e., bilateral cingulate gyri, middle and inferior frontal gyri, fusiform and superior temporal gyri, and bilateral parietal and bilateral occipital lobes). In addition, Diez-Ciranda et al. [213] observed that reduced gray matter volume in the precentral and postcentral gyrus and in the middle and inferior frontal gyri may be involved in ToM deficits in PD. Moreover, these authors reported an association between ToM impairment and alterations of white matter in the superior longitudinal fasciculus, adjacent to the parietal lobe, and of the white matter adjacent to the frontal lobe.

In atypical parkinsonisms, ToM abilities have been poorly explored. In detail, impaired ToM abilities have been described in PSP patients [188] and found to be related to grey matter atrophy in the right inferior frontal gyrus and in the anterior medial frontal cortex, both associated to the ToM domain. Finally, Poletti and Bonuccelli [214] described a case of affective ToM impairment in a case of clinically diagnosed CBS with a bilateral 18-FDG positron emission tomography hypometabolism in the frontal-temporal-parietal cortices, more marked in the right hemisphere. However, no data have been reported about ToM abnormalities in patients affected by multisystem atrophy.

### 2.3. Alzheimer's Disease

Alzheimer's disease (AD), the most frequent neurodegenerative dementia and the first cause of neurocognitive disorder in the world, is typically characterized by an early and progressive episodic memory loss. From the neuropathological point of view, the progressive neurodegeneration initially affects the hippocampi, the entorhinal and posterior cingulate cortices, and, subsequently, the entire temporal, parietal, and frontal cortices [215]. Following this anatomical pathway, social cognition dysfunctions can occur during the course of the disease, particularly in the moderate-severe stages [216]. In contrast, earlier in the disease, social incongruities appear largely modulated by general cognitive decline in memory, language, and executive domains, rather than a genuine social dysfunction [217].

In particular, although considerably less common than in bvFTD, AD patients may present impaired social behavior, ToM, loss of empathy, facial emotion recognition, and inaccurate self-awareness, and, although uncommon as first symptoms, among alterations of social behavior, disinhibition, social awkwardness, and apathy have been reported, respectively, in 6.9%, 5%, and 2% of cases [218].

#### 2.3.1. Alterations in the Four Domains of Social Cognition

With regard to apathy and disinhibition, associated with AD severity, one of the most common tools for social/behavior evaluation in dementia, the Neuropsychiatric Inventory (NPI) [219], has been used in combination with the Clinical Dementia Rating (CDR) scale [220], which is a disease severity scale, ranging from 0 (no impairment) to 2 (severe impairment). Several NPI dimensions, but in particular apathy and disinhibition, were correlated with the CDR score. Moreover, apathy was the most prevalent in all CDR groups and the only symptom with frequencies exceeding 50% in AD patients with CDR 0.5, 1, and 2 [221].

Other types of social disturbances are rarely described in AD, such as criminal behaviors, which are more recurrent in bvFTD [101]. In fact, rarely, AD patients may present an atypical, bvFTD-like clinical profile in very early stages of disease. However, this presentation is characterized by a milder and more restricted behavioral profile than in bvFTD, with high cooccurrence of memory dysfunction and dysexecutive abnormalities, and a pattern of atrophy centered on temporoparietal regions, as in typical AD [222].

Deficits in recognizing others' emotions are reported in AD [223] and in its prodromal stage, the so-called mild cognitive impairment (MCI) [224]. Most emotion recognition studies have required participants to identify emotional expressions in pictures producing mixed results, with evidence of both impaired [223, 225-227] and intact [228, 229] recognition overall. When considering specific emotions, the findings are also inconsistent. More recently, a large sample of neurodegenerative patients, including AD, was studied by using short films instead of photographs [229]. This study revealed that emotion recognition was indistinguishable by comparing the AD group to healthy controls. However, in this regard, it should be kept in mind that concomitant basic and high-level visual and visuospatial difficulties in AD may negatively impact facial recognition, which in turn translate in an impaired emotion recognition [230].

ToM deficits are reported in AD [231, 232], although ToM alterations in AD remains a controversial subject. Specifically, performances at ToM tests have been revealed to not reflect a genuine ToM deficit, rather a deficit mediated by general (and particularly executive) cognitive decline [233].

Loss of empathy has also been reported in AD, particularly as the so-called cognitive empathy (i.e., the ability to understand) in the context of a relative preservation of affective empathy (i.e., the ability to share) [234]. This pattern of spared/impaired types of empathy has been shown to be related to the vulnerability of a distributed network of regions, centered on the frontoinsular cortices, the integrity of which in AD is crucial for preserved social functioning [234].

Finally, AD patients may also present poor self-awareness of their functional limitations that may exacerbate their behavior abnormalities, as well as the reliability of the patient [235]. Self-awareness can be easily tested by asking the patient for a description of themselves, using a scale rating their competency across different domains (i.e., daily living activities and cognitive/emotional interpersonal control) (such as the Patient Competency Rating Scale) [235]. In particular, AD patients resulted in being more prone to overestimate cognitive and emotional functioning in comparison to bvFTD patients who may overestimate their functioning in all domains [235].

## 3. Conclusions and Future Perspectives

Abnormal interpersonal behavior is commonly observed in clinical practice, representing part of the core diagnostic criteria for many clinical disorders. Therefore, to overcome the potential consequences related to social isolation, known to be a risk factor for morbidity and mortality [2], social cognitive intervention may be prospected to reduce the negative impact of such disabilities on mental health, improving the ability to form and consolidate interpersonal networks. Among these strategies, targeted training programs may be implemented on the basis of the recent evidence of structural plasticity in well-known socioaffective and sociocognitive brain networks after training-induced behavioral improvements in healthy adults [236] and of potential advantages from targeted training programs on emotion recognition in neurodegenerative patients [104, 237].

Recently, increasing interest has been addressed to the potential benefits of pharmacotherapy on social cognition deficits, such as the potential effects of the peripheral administration of exogenous oxytocin, shown to exert prosocial effects [238-241], probably mediated by the modulation of the serotonergic system [239].

Another emerging approach is related to the use of brain stimulation techniques, such as theta burst and high-frequency repetitive transcranial magnetic stimulation (rTMS), to modulate empathy-related brain activity. In particular, rTMS sessions, by stimulating the bilateral medial prefrontal cortex, have been revealed to be useful in improving self-reported social functioning in the case of autism spectrum disorders [242] and major depressive disorders [243].

Finally, given the critical role of caregivers' wellbeing and collaboration in any therapeutic and rehabilitation plan, treatment efforts should also be directed towards ensuring the availability of appropriate education and support for them.

## Figures and Tables

**Table 1:** Social cognition domains with the related assessment tools for clinical and research investigation of social cognition deficits in neurodegenerative diseases.

Social cognition domains	Assessment tools	Neurodegenerative diseases with impairment of social cognition domains	Neuroanatomic correlates of social cognition dysfunctions in neurodegenerative diseases
Theory of mind	False-belief tasks, Faux-Pas Test, RMET, Strange Stories Test, TASIT	bvFTD	Ventromedial, dorsolateral, and ventrolateral prefrontal cortex, OFC, temporoparietal junction [42, 43, 115-119]
		nfvPPA	Temporal pole, insular cortex [113]
		svPPA	Left temporal lobe, medial frontal cortex [113]
		ALS	Dorsomedial and dorsolateral prefrontal cortex, supplementary motor areas [137]
		PD	Bilateral cingulate gyri, middle and inferior frontal gyri, fusiform and superior temporal gyri, bilateral parietal and bilateral occipital lobes [212, 213]
		PSP	Right inferior frontal gyrus, anterior medial frontal cortex [188]
		CBS	Right frontal-temporal-parietal cortices [214]
		AD	Superior temporal sulcus, posterior cingulate cortex, precuneus [42]

Empathy	EPT, EQ, IRI-EC, IRI-PT, MET	bvFTD, ALS, AD/MCI	Anterior cingulate, frontoinsular cortices [139, 115-119, 234]

Social perception	Ekman Faces test, TASIT, Comprehensive Affect Testing System, Florida Affect Battery	bvFTD	Anterior cingulate, orbitofrontal and medial prefrontal cortex, insula, striatum, and amygdala (SLN) [42, 43, 109, 115-119]
		nfvPPA	Posterior fusiform gyri, bilateral insular cortex, anterior temporal lobe [112]
		svPPA	Left temporal cortex, amygdala [112]
		ALS	Right inferior longitudinal fasciculus, inferior frontooccipital fasciculus [147, 148]
		PD	Orbitofrontal cortex, right and left superior frontal gyri, bilateral posterior cingulate gyri, somatosensory cortices, amigdala [206-209]
		PSP	Right inferior frontal gyrus [188]
		CBS	Paracentral gyrus, precuneus [163]
		AD	Temporoparietal regions [222-224]

Social behavior	AES, FBI, FrSBe scale, NPI, SDS, SIRS	bvFTD, ALS, PD, AD	Ventromedial and lateral prefrontal cortex, fronto-temporo-insular areas, anterior cingulate cortex [21, 22, 118, 129, 130, 222]

AD = Alzheimer's disease; AES = apathy evaluation scale; ALS = amyotrophic lateral sclerosis; bvFTD = behavioral variant frontotemporal dementia; CBS = corticobasal syndrome; EQ = Empathy Quotient; FrSBe = Frontal Systems Behavior; FBI = Frontal Behavioral Inventory; IRI-EC = Interpersonal Reactivity Inventory-Empathic Concern; IRI-PT = Interpersonal Reactivity Inventory-Perspective-Taking; MCI = mild cognitive impairment; MET = Multifaceted Empathy Test; NPI = Neuropsychiatric Inventory; OFC = orbitofrontal cortex; PD = Parkinson's disease; PSP = progressive supranuclear palsy; RMET = Reading the Mind in the Eyes Test; SDS = Socioemotional Dysfunction Scale; SIRS = Social Impairment Rating Scale; TASIT-S = The Awareness of Social Inference Test.
